# Adolescent girls and young women as a critical population for HIV epidemic control in sub-Saharan Africa: are we doing enough?

**DOI:** 10.3389/fpubh.2025.1670190

**Published:** 2025-11-13

**Authors:** Grant Murewanhema, Enos Moyo, Perseverance Moyo, Tafadzwa Dzinamarira

**Affiliations:** 1 Unit of Obstetrics and Gynaecology, Department of Child, Adolescent and Women's Health, Faculty of Medicine and Health Sciences, University of Zimbabwe, Harare, Zimbabwe; 2 Department of Public Health Medicine, University of KwaZulu-Natal, Durban, South Africa; 3 Nursing Department, Medical Centre Oshakati, Oshakati, Namibia; 4 School of Health Systems and Public Health, University of Pretoria, Pretoria, South Africa; 5 ICAP, Lusaka, Zambia

**Keywords:** adolescent girls and women, HIV, HIV prevention, HIV programs, Africa

## Introduction

Without proper control measures, sub-Saharan African (SSA) adolescent girls and young women (AGYW) aged 15–24 are at high risk of HIV infection. Despite significant progress in the control of the HIV epidemic, most new infections in SSA in 2023 occurred in AGYW. High sexual and reproductive activity in AGYW contributes significantly to the HIV epidemic, making them a priority population for focused prevention efforts that uphold their sexual and reproductive health and rights (1).

Since the 1995 HIV epidemic peak, new infections have plummeted 60%. Still, in 2023, an estimated 1.3 million people were newly infected globally. In SSA, women and girls account for 62% of new infections, compared to 44% globally. AGYW in SSA are particularly vulnerable, with 3,100 new infections occurring weekly (1). HIV prevalence among AGYW aged 15–24 remains significantly higher than among their male peers of the same age group; in some countries, prevalence among young women is more than double that of young men (2). Several factors contribute to this crisis. Gender inequality, especially gender-based violence, hampers AGYW's HIV protection. Sexuality norms and taboos limit their healthcare and decision-making ability. In addition to gender and sexuality norms, other intersecting social norms, such as adultism, which privileges adult authority over adolescent autonomy, and heteronormativity, which marginalizes diverse sexual identities, also shape AGYW's sexual and reproductive health and rights (SRHR) outcomes in SSA. Violence against AGYW is a risk factor for HIV and worsens health outcomes and treatment access (3).

Poorer, less educated AGYW in eastern and southern Africa struggle with condom use, emphasizing inequities. Lack of safe spaces, limiting parental consent, damaging gender norms, punishing laws, and the exclusion of pregnant girls from education impair HIV prevention for AGYW (4). Clinical trials have clearly demonstrated the effectiveness of biomedical interventions such as PrEP, PEP, and condom use in preventing HIV infection; however, their impact is maximized only when accompanied by structural and social interventions that address the broader barriers faced by AGYW (5). Understanding the facilitators and hurdles to preventative measures is crucial for HIV control policy and programming in SSA, as is a review of epidemic control efforts in this region. This opinion paper discusses what needs to be done to control the AGYW outbreak and whether major gaps remain that need redress.

Increased condom access and biological tools like PrEP and PEP have improved HIV prevention for AGYW in SSA. Despite these developments, the epidemic persists, showing response gaps. Medical interventions have promise, but a lack of information, cultural hurdles, and poor healthcare access restrict their impact. Poverty, gender inequality, and early marriage are important social determinants of health, yet biomedical interventions generally take precedence. Evidence from successful gender equality and social norms interventions demonstrates that shifting harmful gender and power dynamics can significantly enhance HIV prevention outcomes (6, 7). For example, the SASA! Community mobilization intervention in Uganda reduced physical intimate partner violence and concurrent sexual partnerships, thereby lowering HIV risk (8). Similarly, interventions promoting gender-equitable norms among adolescents have been associated with increased condom use and reduced risky sexual behaviors. These examples highlight the importance of combining biological and structural interventions to address the underlying drivers of the epidemic. However, top-down programs have often neglected young people's needs, preferences, and challenges, limiting the sustainability of these gains. With this opinion piece, we present multifaceted approaches that combine biomedical and structural interventions that are needed to combat the AGYW HIV epidemic.

## Addressing gender inequality and sexual health

### Gender equality

Gender equality programs and policies should start in early adolescence before gender biased attitudes become entrenched and intractable. Equality and empowerment are essential to minimizing HIV vulnerability in women. This includes challenging harmful gender norms and stereotypes, increasing girls' education, and providing economic opportunities for young women. Nonetheless, it is critical to recognize that gender inequality is a result of numerous structural causes. Therefore, only employing empowerment techniques and behavior modification may not be sufficient (9).

### Sexual and reproductive health education

School curricula should include comprehensive sexuality education (CSE) on abstinence, postponing or minimizing sexual contact, safer sex interventions, HIV prevention, contraception, and gender equality (10). Peer-based education programs, sports-based, internet-based, or a mix of these CSE interventions can be applied in schools and communities (11). In Kenya, a program that included yearly HIV testing days, mobile HIV testing in school settings, and the distribution of messages on abstinence, faithfulness, and condom use to students ranging from primary to university levels improved CSE. Peer co-facilitators or adult facilitators can offer CSE. Additionally, CSE can be delivered through the media, phone conversations, and online information. A systematic review found that technology-based interventions, such as teen-led media literacy curricula centered on media portrayals of sexuality and customized computerized interventions whose content and delivery were based on the Information-Motivation-Behavioral Skills model of health behavior change, were effective in enhancing sexual knowledge (10). Evidence indicates that abstinence-only programs are generally not effective in reducing HIV risk or delaying sexual debut when delivered in isolation. Instead, greater impact is observed when messages on abstinence are delivered as part of a broader package of CSE interventions that include information on contraception and condom use.

### Community engagement and mobilization

AGYW can benefit from community-based gender-based violence, child marriage, and poverty programs. Trade exhibitions, weddings, funerals, and community meetings can be used to engage and mobilize communities. Community engagement and mobilization are essential for effective HIV prevention among AGYW. Beyond raising awareness, community-led approaches play a critical role in generating understanding and acceptability of reproductive health products such as PrEP, while also addressing harmful social norms that limit access to services, including gender-based violence, child marriage, and stigma (12). In the SASA! Cluster randomized control trial in Kampala, Uganda, a community mobilization intervention to prevent violence against women and lower HIV risk was found to significantly reduce concurrent sexual partners and physical intimate partner violence (8).

It is also crucial to acknowledge that while community engagement is vital, communities themselves can reinforce stigma and restrictive gender norms that increase AGYW's vulnerability to HIV. Effective HIV prevention therefore requires dual education, empowering AGYW directly, while also sensitizing communities to recognize and change the social and cultural practices that sustain risk. This kind of widespread change is challenging but essential for sustainable progress.

## Generating adequate trust in biomedical interventions

### Community-led approaches

Communities may build trust and ownership by designing and implementing HIV prevention initiatives. Biomedical preventative interventions like PrEP and PEP necessitate coordinated awareness campaigns. To encourage AGYW to disclose their use of these interventions and improve adherence, these initiatives should focus on parents and community gatekeepers. Parental support may increase AGYW biomedical intervention uptake and adherence (13). Schools, communities, and healthcare institutions can all be the sites of demand generation initiatives for biomedical interventions and awareness campaigns (14).

### Comprehensive and integrated services with decentralized delivery mechanisms

Building trust in biomedical interventions also requires comprehensive service provision, including sexual and reproductive health care, mental health support, and social services. Evidence from Cluver, Toska, and Rudgard demonstrates that addressing these interconnected needs through “accelerator” interventions can improve HIV prevention and treatment outcomes for adolescents in SSA (15). Expanding access further requires decentralized health delivery models, such as mobile health clinics, outreach programs, and community health workers. These approaches can bring PrEP, PEP, and other SRH services closer to AGYW, especially in underserved rural or peri-urban settings. Decentralization has been shown to reduce structural barriers, improve service uptake, and promote continuity of care. For example, AGYW may be more likely to adopt biomedical interventions when they are delivered by their peers. A Ugandan study found that peer delivery, which provides non-judgmental, client-friendly services and adherence support, encourages young women to take HIV self-testing and PrEP and promotes treatment persistence (16).

## Empowering AGYW to make informed decisions

### Life skills education

The capacity for good and adaptable conduct that helps people to successfully navigate the rigors and difficulties of daily life is known as life skills. It helps AGYW develop healthy relationships, clear communication, and responsible decision-making to prevent HIV. A narrative systematic review by Nasheeda et al. (17) found that life skills education enhances decision-making, communication, and problem-solving abilities, but highlighted that the inclusion of mentorship components, particularly age-similar mentors, strengthens program effectiveness (17). Life skills education can therefore be closely linked with mentorship programs, where peer or slightly older mentors provide guidance, support, and role modeling, creating opportunities for AGYW to apply their skills in real-life contexts and make safer choices regarding sexual and reproductive health (18). A South African secondary school study found that learning about life skills and HIV/AIDS boosted students' awareness of the disease, delayed sexual activity, and improved their attitudes toward HIV-positive people (19).

### Youth-friendly services

Creating youth-friendly health services that are accessible, confidential, and non-judgmental is essential. These programs should treat AGYW's social and emotional needs as well as HIV prevention. To build stronger relationships with AGYW, youth-friendly healthcare workers should actively listen and encourage discussion of broader health or life issues outside of HIV prevention. In addition to youth-focused services, AGYW facilities should offer tangible items like internet and menstrual pads (20). Programs should engage with schools on the benefits of condom provision at school to ensure that permission is granted (14).

Many AGYW at high risk of HIV are not abstract populations but real individuals navigating complex economic and social realities. Some engage in transactional or sex work–related activities as a means of survival or to support their families. HIV prevention strategies, including self-testing and PrEP, should therefore acknowledge and respond to these lived circumstances rather than treating them as uniform risk categories. Recognizing AGYW as active agents striving to secure livelihoods and futures helps shift the focus from vulnerability to agency. This perspective ensures that interventions are grounded in empathy, respect, and the realities of their daily lives.

### Mentorship programs

Support and guidance from mentorship programs can help AGYW overcome barriers and make sensible decisions. AGYW can gain confidence, self-worth, and academic success, and exhibit fewer deviant behaviors in mentoring programs with peers or stable, caring, non-parental adults (21–23). A Ugandan study found that peer mentorship increased HIV/AIDS knowledge, beliefs, and prevention attitudes at 12-month follow-up (24).

### Economic empowerment

Financial empowerment can assist AGYW in preventing HIV infection. Financial empowerment can involve monetary transfers or projects. A systematic review by Stoner et al. (25) highlights that cash transfer programs are effective in improving school attendance and reducing HIV risk behaviors among adolescent girls (25). Similarly, a Ugandan study that combined savings-led economic empowerment through youth development accounts and evidence-based family strengthening intervention delivered via multiple family groups found that AGYW were motivated to participate because of the promised matched savings (26). To prevent AGYW from having age-disparate or transactional sex, which can hinder condom negotiation, programs should scale up economically strengthening interventions such career counseling, academic support, and job opportunities.

The disproportionate burden of HIV among AGYW in SSA is a critical public health issue. While progress has been made, significant gaps remain in the prevention landscape. Addressing these challenges requires a comprehensive approach that combines biomedical interventions with structural interventions to address the underlying social determinants of health.

Despite clear evidence on the effectiveness of multi-component interventions, ranging from biomedical tools like PrEP and PEP to structural approaches such as gender equality programs, comprehensive sexuality education, mentorship, and economic empowerment, implementation remains uneven. Many aid agencies and governments recognize the importance of these broader responses, and programs such as PEPFAR's DREAMS initiative and the Global Fund for TB, AIDS, and Malaria have actively supported multi-component approaches. However, reductions in funding from donor agencies and shifting priorities threaten the continuity and scale-up of these interventions, limiting their reach and impact. Sustainable HIV prevention for AGYW will therefore require not only evidence-based programming but also renewed investment and political commitment to ensure that multi-component interventions can be implemented consistently and at scale.
